# Supplementary material for: Human TRMT2A methylates tRNA and contributes to translation fidelity
Source: Nucleic Acids Res. 2023 Jul 3;51(16):8691–710. doi: 10.1093/nar/gkad565 (PMC10484741; doi:10.1093/nar/gkad565)
Supplement: gkad565_Supplemental_Files [file gkad565_supplemental_files.zip › Supplementary_table_legends.pdf]

Supplementary Tables

Supplementary Table 1: **RNA sequences produced by in vitro transcription**

Supplementary Table 2: **List of primer used for cloning and mutagenesis**

Supplementary Table 3: **Results from crosslinking and mass spectrometry experiments**

Supplementary Table 4: **Proteins significantly enriched/depleted in BioID data**

Supplementary Table 5: **Proteins significantly enriched/depleted in Co-IP data**

Supplementary Table 6: **Instrument parameters for LC-MS/QQQ analysis of nucleosides**
